# Perforated Neonatal Appendicitis with Pneumoperitoneum

**Published:** 2013-05-27

**Authors:** Yousuf Aziz Khan, Kanwal Zia, Nasir Salim Saddal

**Affiliations:** Department of Paediatric Surgery, National Institute of Child Health (N.I.C.H.), Rafiquee Shaheed Road, Karachi – 75510, Pakistan.

**Keywords:** Pneumoperitoneum, Neonatal appendicitis, Mortality

## Abstract

Acute appendicitis is a rare condition in neonates, with a high mortality. If perforated, it seldom presents with radiologically significant pneumoperitoneum. An 11-day-old newborn presented with abdominal distension and reluctance to feeds. X-ray abdomen revealed significant pneumoperitoneum. After optimization of his condition, exploratory laparotomy was performed. Perforated appendix was found and appendicectomy done. Post operative course was stormy that lead to demise of the baby.

## INTRODUCTION

Neonatal appendicitis (NA) is a rare clinical entity. It has a low incidence of 0.04 – 0.2% and is more common in premature male babies.[1] Less than 50 cases have been reported over the last 30 years, with just above 100 over the last century.[2] Neonatal appendicitis usually presents with non-specific symptoms and signs. Because of its rarity and low index of suspicion, it poses a great diagnostic challenge pre-operatively. The resultant delay in diagnosis and its complications like perforation and septicemia confers a high mortality rate of 28%.[3] Herein, we report a case of a newborn who was found to have perforated appendix at laparotomy.

## CASE REPORT

An 11-day-old male newborn weighing 2.4 kg was referred with abdominal distension and reluctance to feeds for 8 days. Antenatal history was insignificant. He was delivered at full term via Caesarean section. There was no history of birth asphyxia, cyanosis or seizures. Few hours after birth, he developed respiratory difficulty and admitted to NICU at a local hospital. He had passed urine and normal meconeum in time. Expressed breast milk was given via NG tube while he was at NICU. Laboratory results were within normal limits except leucocytes count of 14400/mm3 with 76% neutrophils and, x-ray abdomen was unremarkable. He received IV fluids and broad spectrum antibiotics. There was no history of fever and vomiting. Despite treatment, his condition worsened. Baby developed gradual abdominal distension and was shifted to another hospital. His repeat laboratory tests showed total leukocyte count of 7,900/mm3 and platelets count of 31,000/mm3. X-ray abdomen showed signs of significant free intra-peritoneal air and ultrasound abdomen was inconclusive. He was then referred to our hospital. 

On arrival at our facility, the newborn had a heart rate of 126 beats/min and respiratory rate of 38 breaths/min. He had mild dehydration. Abdomen was distended and tense. X-ray abdomen revealed significant pneumoperitoneum. Tube laparostomy was performed initially, which drained free air and only few ml of turbid fluid. After optimization of his parameters, exploratory laparotomy was performed at the 3rd day of admission. Hyperemic but normal consistency small bowel loops covered with flakes of pus were noticed. The whole colon was of normal caliber. A small perforation was found at the tip of appendix (Fig. 1). Appendicectomy was performed. Post operatively, despite aggressive treatment his condition did not improve and expired on 7th post operative day. Histopathology of the appendix showed normally innervated appendix, with polymorphonuclear infiltration into serosal and muscle layers confirming the diagnosis of acute appendicitis.

**Figure:**
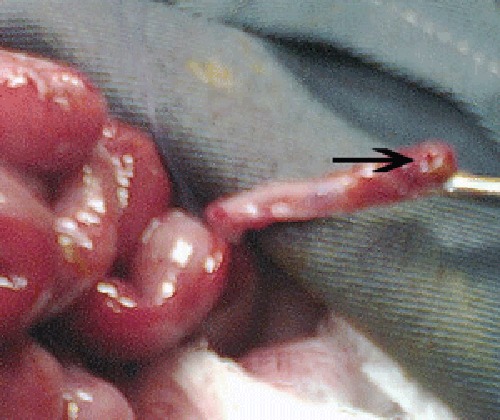
Figure 1: Perforated appendix at the tip.

## DISCUSSION

Neonatal appendicitis is extremely rare condition. Some authors consider it a secondary pathology, developing as a complication of an underlying disease (e.g. Hirschsprung’s disease, cystic fibrosis), while others consider it as a localized form of necrotizing enterocolitis (NEC), limited to the appendix.[4, 5] The theory of localized NEC supports the pathology in our patient as no other cause could be identified. The newborns with early NA usually present with non-specific symptoms and signs. Abdominal distension however, is usually the most common presenting feature.[2] Plain abdominal roentgenograms are usually insignificant in initial stages. The vague clinical features along with low index of suspicion usually result in the delay in diagnosis, predisposing to risk of perforation which is reported to be as high as 80%.[6,7] Similar was the presentation in the index case. 

As the disease progresses, respiratory difficulty, temperature instability, abdominal wall changes, a palpable abdominal mass, signs of sepsis may add to the clinical findings. Rarely could pneumoperitoneum be demonstrated on plain roentgenogram as was present in our case. Pneumoperitoneum has a reported occurrence of only 8% in the cases of perforated appendicitis.[8] Plain abdominal x-rays, ultrasound and CT scan abdomen all have been advocated in the radiological workup of a suspected NA, with CT scan abdomen being the most sensitive and specific tool.[2] Treatment aims at early recognition and appendectomy. 

In conclusion, involved clinicians should be aware of varied presentation of this lethal condition, must keep a high index of suspicion and include NA in the differential diagnoses. Using sophisticated modalities where available and early surgical intervention when the diagnosis is in doubt, may provide a favorable outcome.

## Footnotes

**Source of Support:** Nil

**Conflict of Interest:** None declared

